# StaphSCAN: a genomic surveillance framework for *Staphylococcus aureus*

**DOI:** 10.1186/s12864-026-13165-0

**Published:** 2026-07-09

**Authors:** Riccardo Bollini, Valeria Cento

**Affiliations:** 1 Department of Public Health, Experimental and Forensic Medicine, University of Pavia, Pavia, Italy; 2 Department of Biomedical Sciences, Humanitas University, Pieve Emanuele, Italy; 3 Microbiology and Virology, IRCCS Humanitas Research Hospital, Rozzano, Italy

**Keywords:** Whole-genome sequencing, Bacteria, Surveillance, Bioinformatics, Staphylococcus aureus, MRSA

## Abstract

**Background:**

*Staphylococcus aureus* is a leading cause of hospital- and community-acquired infections. While Whole Genome Sequencing (WGS) has become the gold standard for surveillance, extracting actionable epidemiological data typically requires assembling fragmented workflows of disparate software tools. We introduce StaphSCAN, a modular, open-source Python tool designed to streamline *S. aureus* genomic analysis.

**Results:**

StaphSCAN integrates essential typing methods (MLST, *spa* typing, SCC*mec* typing, capsular typing) with the detection of antimicrobial resistance (AMR), virulence, and biofilm-associated genes. It produces a comprehensive, normalized tabular report suitable for immediate interpretation. We validated StaphSCAN using a public dataset of 404 clinical *S. aureus* genomes collected in 2019 during a study conducted by the StaphNET-SA network. The tool successfully characterized the population structure, replicating the main key findings, demonstrating high throughput and reliability.

**Conclusions:**

In conclusion, StaphSCAN provides a lightweight framework for *S. aureus* genomics, facilitating rapid genomic surveillance.

**Supplementary Information:**

The online version contains supplementary material available at https://doi.org/10.1186/s12864-026-13165-0.

## Introduction

*Staphylococcus aureus* remains a high-risk pathogen in both healthcare and community settings, responsible for a wide spectrum of diseases ranging from skin and soft tissue infections to life-threatening bacteraemia and endocarditis [1, 2]. The global burden of *S. aureus* is worsened by the persistence of Methicillin-Resistant S. aureus (MRSA) and the emergence of hypervirulent clones [1, 3]. Consequently, precise epidemiological surveillance is essential to monitor clonal dynamics, detect outbreaks, and track the evolution of antimicrobial resistance (AMR) and virulence determinants [4].

Whole Genome Sequencing (WGS) has revolutionized this surveillance, offering unparalleled resolution compared to traditional typing methods [5, 6]. However, the translation of WGS data into public health action is often limited by the complexity of bioinformatic analysis [7, 8]. A typical workflow for *S. aureus* usually requires several tools, often leading to software conflicts and generation of non-standardized, hard-to-read, outputs. While recent efforts have begun to address this gap by developing species-specific pipelines [9], these solutions can still be computationally intensive, produce “general’ results, and generate highly fragmented output files that complicate large-scale data aggregation. While comprehensive lightweight frameworks exist for other pathogens, most notably Kleborate [10] for *Klebsiella pneumoniae*, a similar tool focused on *S. aureus’* genome is still lacking. To bridge this gap, we developed StaphSCAN, a modular Python-based framework designed to act as an “all-in-one” typing tool for *S. aureus*, focused on clinical-relevant genes. It integrates species identification, assembly quality control, and extensive genotyping (MLST, spa, SCCmec, capsule) with the screening of AMR, biofilm, and virulence genes, and integrates all the results into a single, ready-to-use report. In this article, we describe the implementation and architecture of StaphSCAN. We demonstrate its utility and performance by applying the tool to analyse clinical isolates from a South America study [11] by StaphNET-SA network.

## Implementation

### StaphSCAN implementation

StaphSCAN (v0.4.0) is implemented in Python (v3.10+), and it is designed as a modular, command-line tool. It prioritizes ease of deployment and minimal dependency conflicts, relying on a lightweight stack of core bioinformatics tools: Mash [12] for distance estimation and BLAST [13] for local alignment.

It works on assembled genomes (FASTA format), typing each one against a set of databases, and reporting all results together in a tab-delimited (.tsv) text file. By default, StaphSCAN will run all available modules, unless specified by --modules.

The output file is structured to resemble the one provided by Kleborate [10], to keep consistency with an already widely used tool.

A comprehensive overview of all modules is provided in Table 1.

**Table 1:** Comprehensive overview of StaphSCAN (v0.4.0) typing modules, targets, methodology, and epidemiological applications

Module	Target genes	Detection	Method	Aim	Database	Reported output
Assembly	Whole genome	Mash distance	Whole genome comparison	Assess minimum assembly quality	Curated RefSeq database	*S. aureus*
MLST	*arcC*,* aroE*,* glpF*,* gmk*,* pta*,* tpi*,* yqiL*	Sequence variation	Blast on housekeeping genes	Lineage assignment	PubMLST database	Ex. ST22
*spa* typing	*spa* gene	Variable Number Tandem Repeats	Targeted repeat detection	Epidemiological surveillance	Ridom SpaServer	Ex. t1022
*agr* typing	agr operon (*agrB*, *agrD*, *agrC*, *agrA*)	Sequence variation and frameshift mutations	Blast	Typing quorum-sensing systems involved in virulence regulation	Curated database	Ex. *agr I*
Capsule typing	*cap* operon	Gene presence/absence	Blast	Capsular completeness and serotyping	Curated database	Ex. Type 5
SCCmec typing	*mec* gene complex & *ccr* recombinase complex	Gene presence/absence and sequence variation	Blast	Cassette classification	Curated database	Ex. Type IVa
AMR	*AAC(6’)-Ie-APH(2’’)-Ia; ANT(4’)-Ia; ANT(4’)-Ib; APH(3’)-IIIa; ANT(6)-Ia; ANT(9)-Ia; mecA; mecC; blaZ; blaZ mecC-type; cfrA; poxtA; ermA; ermB; ermC; ermT; msrA; tetM; tetK; vanA; vanB*	Presence/absence, sequence variation, and point mutations	Blast	Detection of acquired antimicrobial resistance genes / mutations and sequence integrity	CARD-curated database	Ex. mecA; tetM
Virulence	*etA; etB; etD; etE; lukD; lukE; lukF; lukS; sea; sec; she; sell; selk; selq; tsst-1*	Presence/absence and truncation detection	Blast	Assessing pathogenic potential and clinical severity risk	VFDB-curated database	Ex. PVL
Biofilm	*icaADBC; clfAB; fnbAB; cna; icaR*	Presence/absence and truncation detection	Blast	Assessing potential biofilm production	VFDB-curated database	Ex. icaABDCD

#### Taxonomic assignment and assembly quality

- The assembly module (-m assembly) includes a step of taxonomic assignment and a step of quality control.
- The species identification is based on Mash [12] distances between the input genome and a curated dataset of reference genomes. A minimum mash distance of 0.04 with one of the genomes in the database is required to report the specie.

The quality control is based on the following metrics: (i) total genome size, (ii) contig N50, and (iii) number of ambiguous bases (Ns). Whenever the genome size exceeds the expected range of 2.6–3.1 Mbp, or N50 is below 10,000 bp, or ambiguous bases are detected, the quality check is flagged as failed.

#### Typing

StaphSCAN includes five typing modules, each one can be run as a stand-alone module.

The Multi Locus Sequence Typing module (-m mlst) is based on the seven-locus typing scheme [14], as described on the PubMLST website (https://pubmlst.org/organisms/staphylococcus-aureus). For each locus, exact matches, novel sequences, partial matches (i.e. coverage less than the reference allele), or missing matches are reported. Moreover, if no exact ST match is found, the closest one is reported, followed by the number of differing loci (i.e. ST1-1LV, closest match is ST1, with 1 locus variant). Due to new PubMLST policies, it was not possible to redistribute alleles and profiles generated after December 2024, so StaphSCAN come with data up to that date, and each user can update it using the --mlst_update command using its own PubMLST account.

The spa module (-m spa) is used to characterize the repeated regions of the *spa* protein [15]. It is based on the Ridom database (https://spaserver.ridom.de/). Exact matches are reported, as well as novel patterns, or unknown repeated regions.

The agr module (-m agr) identifies the accessory regulator (agr) type, a quorum-sensing system involved in virulence regulation, and it is classified into four major groups [16]. It has been adapted from the tool AgrVATE [16], and re-written to be integrated.

The capsule module (-m capsule) identifies the Staphylococcus aureus capsular polysaccharide operon and assigns the predominant capsule serotype (Type 5 or Type 8). All capsular loci are identified (capA-P), and used to determine the operon completeness, while serotype is inferred based on the serotype-specific loci (capH-K) [17].

The sccmec module (-m sccmec) detects and classifies Staphylococcus aureus SCCmec elements, which carry methicillin resistance determinants and are defined by combinations of the mec gene complex and ccr recombinase genes [18]. The module supports the classification of fifteen different SCCmec types, and twenty-two subtypes. It has been adapted from the tool *SCCmec* (https://github.com/rpetit3/sccmec), and re-written to be included in the pipeline.

#### Biofilm

The biofilm module (-m biofilm) identifies key Staphylococcus aureus genes involved in biofilm formation, including surface proteins (*clfA*,* clfB*,* fnbA*,* fnbB*) [19–21], the collagene-binding protein *cna* [22], the polysaccharide intercellular adhesin (PIA) genes (*icaA–D*) [23–25], and the *icaR* regulator [26]. The genes are detected using nucleotide BLAST [13] search against a database of reference sequences. To summarise the biofilm potential of each isolate, a score is assigned as follows: 0: incomplete or absent *ica* or *fnb* genes; 1: only the *ica* or the *fnb* genes are present; 2: *ica* or *fnb* genes and one between *cna* and *clf* are present; 3: both *ica* and *fnb* genes are present, or one of the two plus both *cna* and *clf* genes; 4: *ica* genes, *fnb*, and *cna/clf* genes are present; 5: all key genes are present. A minimum identity of 90% and coverage of 80% is required to report the hit, with all weak hits (80–90% identity or 40–80% coverage) reported as “spurious” and excluded from the score evaluation. Truncations are detected by translating the nucleotide sequence into amino acids and comparing it to the reference amino acidic length.

The assignment of the biofilm score (0–5) is based on the progressive accumulation of genes driving distinct functional phases of *Staphylococcus aureus* biofilm formation and host colonization. A fundamental role is played by the production of the PIA, by the *ica* genes [25]. The presence and expression of the *ica* operon directly correlate with biofilm-forming phenotype, as deletion or inactivation of these genes results in severely impaired biofilm formation [23]. Given the basic role played, and the common prevalence (28–51% in clinical isolates [24]), its presence was assigned to a score of (1) However, *Staphyloccocus aureus* can produce a PIA-independent biofilm mediated by the *fnbAB* genes [20]. To increase the score, we evaluated the presence of *cna* or *clf* genes in addition to the *ica* or *fnb* operon. *Cna* and *clf* are adhesins, member of the MSCRAMMs family, involved in surface attachments [19, 22]. Their presence, together with the *ica* or *fnb* operon, represents the genotype of a strain capable of attaching a surface and forming a mature biofilm, and for this reason it was given a score of (2) The presence of *fnb* genes alongside the *ica* operon increases the score to 3, representing a functional improvement. Indeed, *fnb* not only facilitate surface adhesion, but are the primary invasins required for *S. aureus* internalization into non-phagocytic host cells via αβ-integrins [21], protecting the bacteria from immune clearance and antimicrobial therapy. Consequently, the score increases to 4 if one between *clf*/*cna* is present alongside *fnb* and *ica*, with the maximum score of 5 assigned to the presence of all the targeted genes. This score reflects the multi-factorial genotype of the strain, capable of colonization, diverse host-protein attachment, intracellular evasion, and redundant biofilm mechanisms.

#### Detection of antimicrobial resistance determinants

The AMR module (-m resistance) screens for known acquired antimicrobial resistance genes using a custom database, that integrates curated entries from CARD (v4.0.1) (https://card.mcmaster.ca/download) and KEGG (https://www.genome.jp/kegg/kegg2.html) databases. Genes are identified using nucleotide BLAST [13], and the best hit is reported. A minimum identity of 90% and coverage of 80% is required to report the hit, with all weak hits (80–90% identity or 40–80% coverage) reported as “spurious” and excluded from the score evaluation. Gene sequences with imperfect identity (< 100%) are translated and aligned to the reference protein; hits resulting in synonymous mutations are retained, while those leading to truncations are reported in a separate column and excluded. Chromosomally encoded mutations associated with AMR are reported, including the fluoroquinolone resistance mutations [27] in GyrA (S84L, E88K, and E88G), GyrB (T451S), and Parc (S80F/Y and E84K/G/V), and the rpoB mutations (H481Y, H481N, L466N, A473T, A477T, S486L) involved in rifampin resistance [28]. A resistance score is inferred to summarize the resistance profile of the isolate, and it is calculated as follows: 0: no beta-lactamases detected; 1: penicillin resistance; 2: methicillin-resistance; 3: vancomycin resistance.

#### Detection of virulence determinants

The virulence module (-m virulence) screens for the genes encoding the main toxins involved in *Staphylococcus aureus* pathogenesis, including tissue invasion and systemic damage [29]. The same parameters used in the resistance module apply. The virulence score summarises the ‘pathogenic’ potential of an isolate, and it is assigned as follows: 0: no target virulent genes found; 1: staphylococcal enterotoxin (*se*) or *lukED* identified; 2: exofoliative (*et*) toxins identified; 3: *tsst-1*/PVL positive.

The scoring system has been designed to quickly categorize the isolates based on a hierarchy of clinical severity and epidemic potential. The score of 1 is associated with the detection of staphylococcal enterotoxins and/or *lukED*. Staphylococcal enterotoxins are located on mobile genetic elements, responsible of food poisoning and massive T-cell activation [30, 31]. *LukED* is a leukotoxin which targets and attacks leukocytes [32], impairing immune system and allowing *Staphylococcus aureus* proliferation [33]. The next score of 2 is related to presence of the *et* toxins, responsible of the staphylococcal scalded-skin syndrome (SSSS), a severe and highly contagious skin disease caused by *Staphylococcus aureus* infection [34]. The last scores of 3 is associated with toxins of high-clinical interests, capable of causing potential life-threating infections. The *tsst-1* gene encodes for a toxin responsible of the toxic-shock syndrome (*tss*), a severe and potentially life-threatening condition [35]. Once thought to be carried mainly by MSSA strains, it has been shown a raise in its frequency in clinical MRSA isolates [36, 37]. PVL, encoded by *lukF* and *lukS*, plays a key role in bacterial virulence [29], binding leukocytes, leading to pore formation and granule release, and contributing to tissue necrosis [38]. It is also associated with skin and soft tissue infections (SSTIs), lung infections (e.g. necrotizing pneumonia) [39], and other non-dermatological conditions like severe musculoskeletal infections such as osteomyelitis, pyomyositis and septic arthritis [29]. Moreover, it has been shown a fitness advantage in PVL-positive strains in community settings [40], with outpatient clinics as the primary entry point for community-acquired (CA) MRSA, contributing to the emergence and dissemination of virulent, antibiotic-resistant strains into hospital settings [41].

### Genomic analysis

We validated StaphSCAN (v0.4.0) by reproducing key findings from a large-scale South America genomic surveillance study by the StaphNET-SA network [11]. Reads were generated by Illumina HiSeq platform with 150 bp paired-end reads, as reported in the study. All raw reads were filtered with Fastp [42] (v0.23.4) (-q 25) and assembled with Shovill (https://github.com/tseemann/shovill) (--assembler spades). The full list of genomes, including database accessions, isolate information, and StaphSCAN results are provided in Supplementary Table S1.

We also validated the reliability of the biofilm score against the publicly available dataset by Noumi et al., Journal of Genetic Engineering and Biotechnology (2025). This dataset was specifically selected because it includes strictly paired in silico genomic data and in vitro phenotypic assay results, specifically microtiter plate (MtP) abiotic biofilm formation and Hep-2 cellular biotic adherence assays.

### Comparative analysis and benchmark

In the process of writing this manuscript a very similar tool, StaphScope [9], has been published. For this reason, we have compared StaphSCAN against it, summarized the features of both tools, and compared their performance on the dataset used in this study. Moreover, we have compared it against the web-based platform Pathogenwatch (v.81) (https://pathogen.watch/en) .

## Results and discussion

We aimed to develop a tool capable of rapid genotyping of *Staphylococcus aureus*, to support from local surveillance to global epidemiology. For this reason, we included taxonomic assignment to species, to lineages via MLST, detection and genotyping of spa, agr, and capsular genes, identification of relevant virulence and AMR determinants (mutations and acquired resistance genes). We tried to include only genomic features with an associated phenotype of clinical relevance, with the inclusion of scores allowing for an immediate interpretation of the presumptive clinical risk associated. All features are summarized in Table 2.

**Table 2:** StaphSCAN (v0.4.0) features

Feature	Description
Identification	Species identification
Assembly quality	N50, ambiguous bases, genome size
Typing	- MLST - spa type - agr type - capsule type - sccmec type and subtype
Acquired Resistance	- Total gene found - Number of drug classes - Alleles grouped by drug class - Truncation - Mutations (gyrA/gyrB/parC, rpoB, 23 S)
Resistance Score	0 = no blaZ/mec/van; 1 = penicillin resistance; 2 = MRSA; 3 = VRSA
Acquired virulence	- Staphylococcal Enterotoxin (se) - Leukocidin (lukED) - Exofoliative toxins (et) - Toxic Shock Syndrom Toxin (tsst-1) - Panton-Valentine (lukFS) - Truncation
Virulence score	0 = none of the above virulence genes ; 1 = se/lukED; 2 = et; 3 = tsst-1/pvl
Biofilm genes	- Ica genes (icaABCD) - Clumping factor gene (clfAB) - Fibronectin Binding Protein (FnbAB) - Collagen-binding adhesin (cna) - Truncation, including the regulator icaR
Biofilm score	0 = none of the above genes; 1 = icaABCD/fnb; 2 = IcaABCD/fnb + cna/clf; 3 = icaABCD + fnb or icaABCD/fnb + cna + clf; 4 = icaABCD + fnb + cna/clf; 5 = icaABCD + clf + cna + fnb

For the species identification, we adopted a mash-based approach, using a curated genome database. We validated it on a set of 45 genomes, including six different staphylococcal species (Supplementary Figure S1, Supplementary Tabel S2).

### StaphSCAN validation

We analysed all the *Staphylococcus aureus* genomes deposited in the ENA archive by the StaphNET-SA surveillance study (415 genomes). Eleven genomes were not included in the original study, and so were excluded from further analyses. Among the remaining 404 genomes, all were correctly identified as *Staphylococcus aureus*, and passed the quality check. A detailed comparison of the results reported by StaphSCAN vs. those reported in the StaphNET-SA network study is provided in Supplementary Table S3.

StaphSCAN’s modules produced results in concordance with the one reported in the original study (Table 3), with the main differences due to the more recent database used by StaphSCAN.

**Table 3:** StaphSCAN (v0.4.0) concordance result

Module	Concordance
Species identification	100% (404/404)
MLST	99.3% (401/404)
Capsular type	100% (404/404)
Spa type	94.1% (380/404)
SCCmec type	99.5% (402/404)

Resistance module was highly concordant with the data reported in the original study, with a 97.5%, 100%, and 100% of concordance for the detection of blaZ, mecA/C, and vanA/B, respectively. Moreover, given the importance of a correct assignment of MRSA/MSSA, when reported, the gene presence/absence of mecA/C was also compared, when provided, to the reported phenotypic interpretation for Oxacillin, with an overall concordance of 99.9% (269/271), a sensitivity of 98.25% (111/113) and a specificity of 100% (158/158). A more detailed analysis, with all the reported AMR genes, is provided in Supplementary Table S4.

**Fig. 1:**
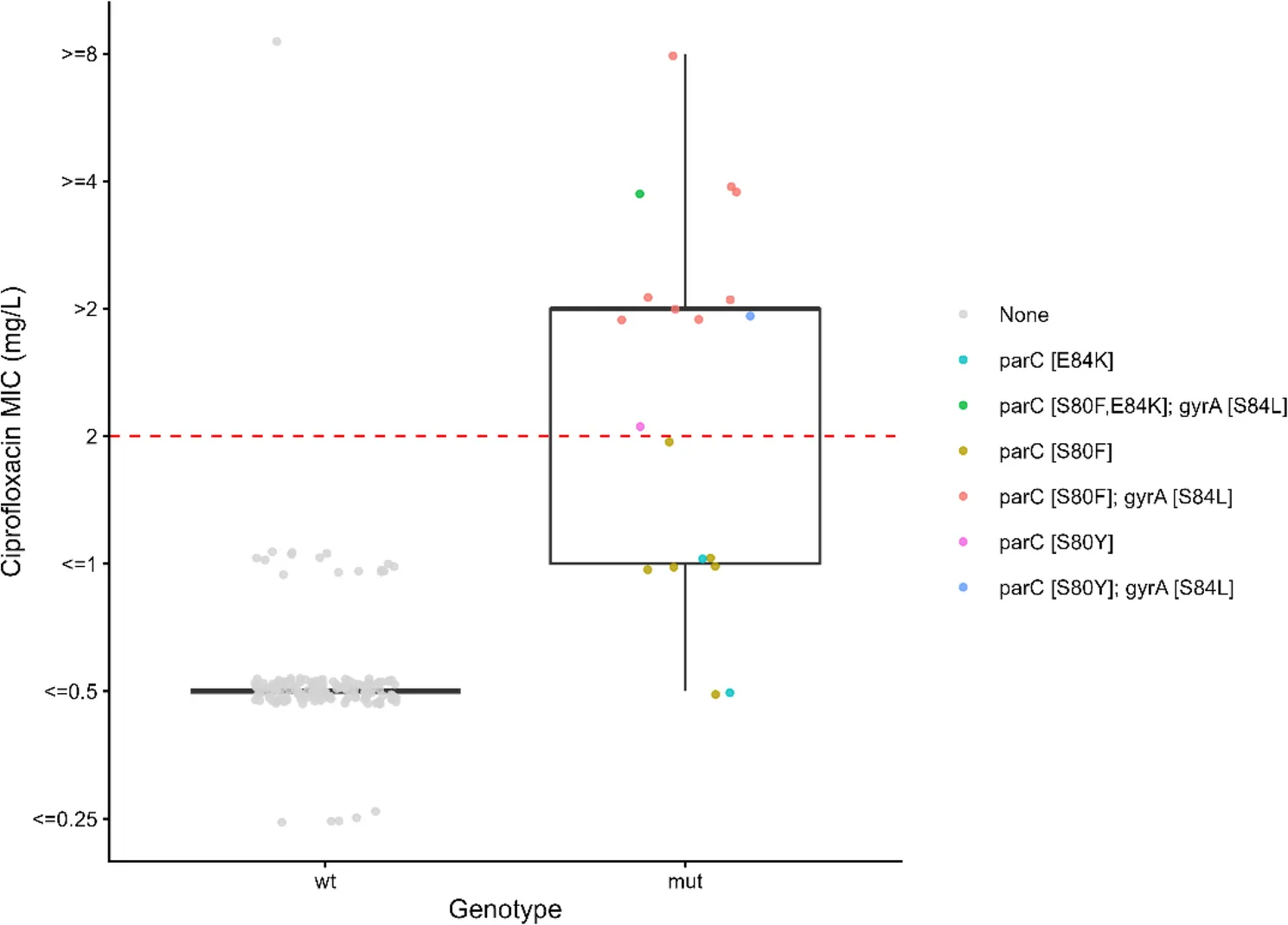
Distribution of Ciprofloxacin Minimum Inhibitory Concentration (MIC), stratified by mutation identified. Each circle represents the reported MIC for an isolate, coloured by the type of mutation identified (if any). Isolate are stratified in wild-type (wt) and mutated (mut) based on the detection of any mutation in one of the target gene (gyrA, gyrB, parC). Dashed line indicates the EUCAST breakpoint for clinical resistance (MIC > 2, EUCAST v.16.0)

In addition to gene presence, StaphSCAN identified also clinical-relevant mutations, contributing to antimicrobial resistance, including the *gyrA*, *gyrB*, and *parC* mutations involved in the Fluoroquinolones acquired resistance [27]. In the dataset, *parC* mutations were the most common but alone did not determine an acquired resistance to Ciprofloxacin, requiring an association with mutation in *gyrA* (Fig. 1).

We investigated also the Rifampin resistance, by analysing the *rpoB* mutations. From the dataset, it was not possible to use the mic values, but instead the categorical values (S or R). For all the isolates (10/10) with a reported *rpoB* mutation by StaphSCAN, the categorical value was ‘Resistant’ (Supplementary Table S5), and the reported mutations in the original study were concordant. For one isolate, with a categorical value of ‘Resistant’, no mutations were reported neither by StaphSCAN or by the original study.

**Fig. 2:**
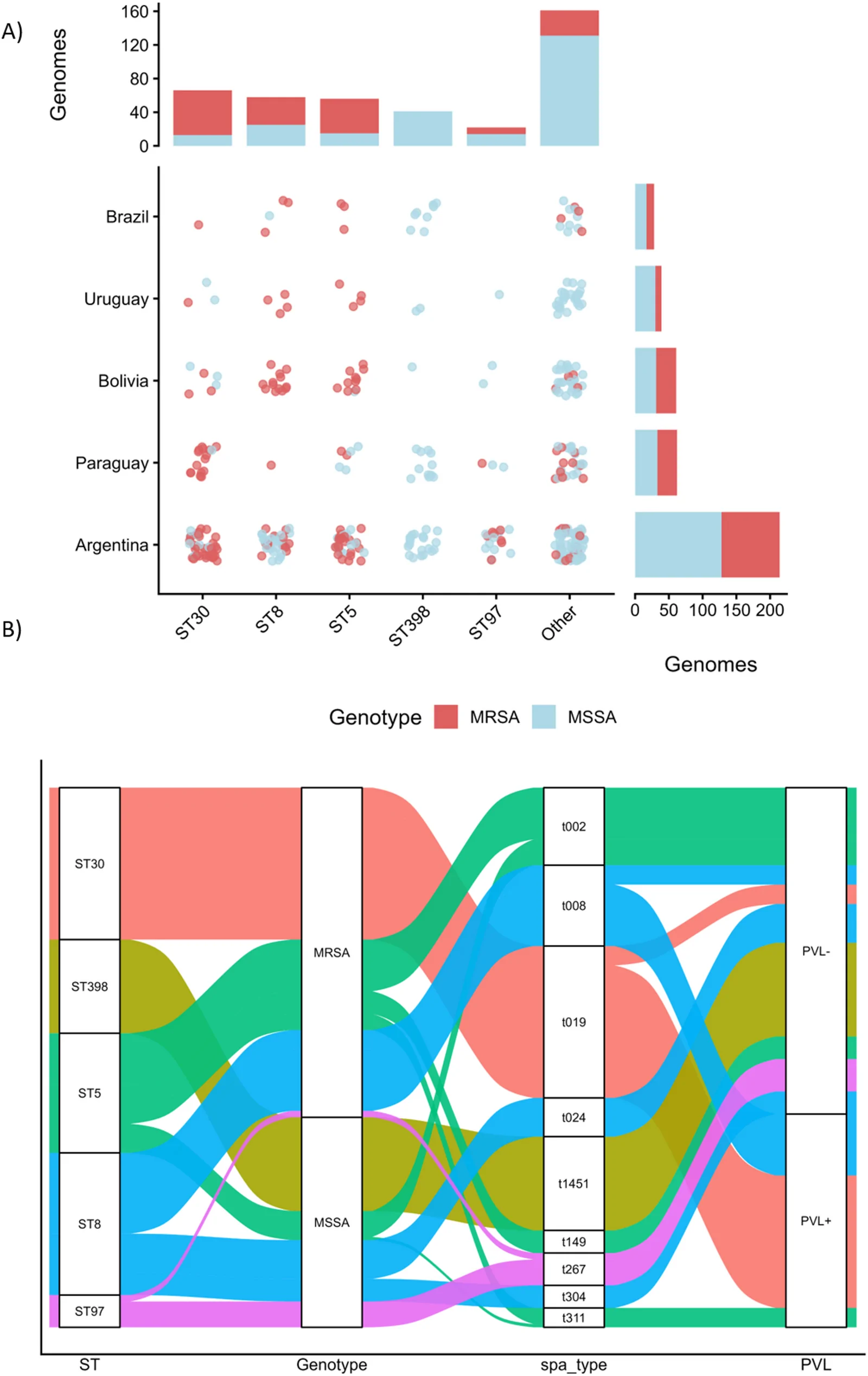
Data shown summarize StaphSCAN results for the 404 genomes from the StaphNET study **A** Geographical and lineage distribution of methicillin-resistant isolates. Each circle represents a genome, coloured by methicillin resistance. Barplots summarize the number of genomes from each lineage (top) and country (right), coloured by methicillin-resistance. All lineages with less than 20 genomes were grouped together under ‘Other’ **B** Genotype summary of the main (*n* > 20) STs. The main molecular markers *for Staphylococcus aureus* epidemiology have been included. Only spa types with a frequency higher than 5 have been represented

The main findings of the original study were also reproduced, including the MRSA prevalence (40.8%, 165/404), the widespread diffusion of the lineages ST30, ST5, ST8, and ST398, their geographical distribution, and their genotypes (Fig. 2A and B).

### StaphSCAN scores

**Fig. 3:**
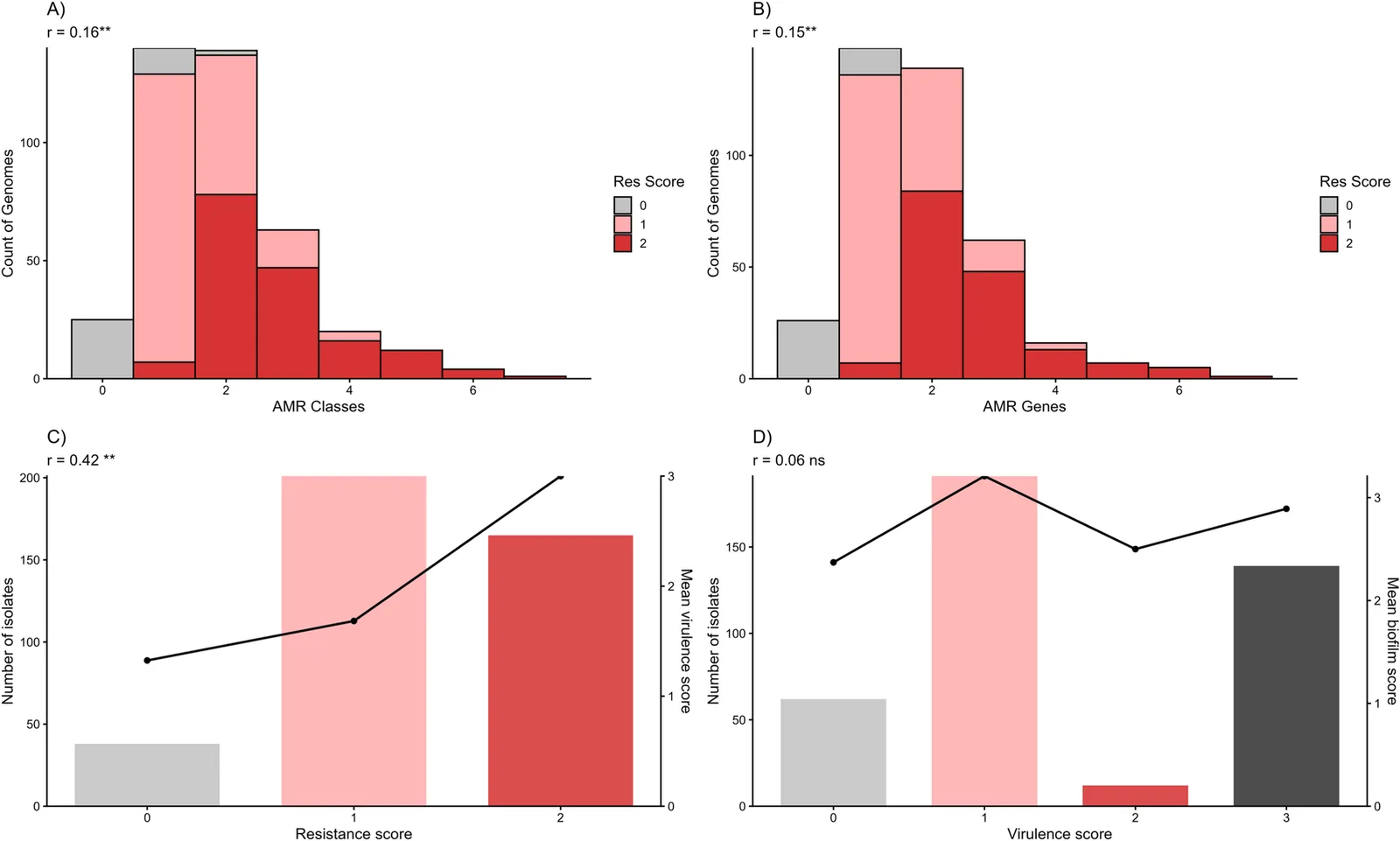
StaphSCAN score summary **A** Histograms showing total number of acquired AMR classes predicted per genome, stratified by resistance score **B** Histograms showing a total number of acquired AMR genes detected per genome, stratified by resistance score. The Spearman correlation coefficients between the resistance score and the number of AMR genes/classes are reported (** *p* < 0.01) (excluding genes/classes contributing to the score) **C** Bar plot represents prevalence of isolates with a specific resistance score. Line plot represents the mean virulence score stratified by resistance score **D** Bar plot represents prevalence of isolates with a specific virulence score. Line plot represents the mean biofilm score stratified by virulence score. The Spearman correlation (r) between the scores is reported (** *p* < 0.01; ns = not significant)

Genomes are scored according to the resistance, virulence and biofilm loci identified. We found that resistance score is not only associated with the specific presence of components contributing to the score, but also with the number of genes and classes. For this reason, genomes with a score higher than 0 usually carry additional genes conferring resistance to multiple drug classes (Fig. 3A and B). Moreover, virulence and resistance scores showed a correlation with each other in the analysed datasets, but none with the biofilm score. As a result, isolates with a higher resistance score tends to have a higher virulence score (*r* = 0.42, *p* < 0.001) (Fig. 3C and D).

The biofilm score has been validated on a dataset of publicly available genomes (*N* = 8), used for the study by Noumi et al., Journal of Genetic Engineering and Biotechnology, 2025.

We compared our in silico genotypic results against their published in vitro phenotypic data (MtP abiotic biofilm and Hep-2 biotic adherence assays). The biofilm scores demonstrated general concordance with the in vitro abiotic biofilm formation capabilities. The model identified the most robust biofilm producer (strain S20), while strains assigned the baseline score of 2 consistently demonstrated low-grade abiotic biofilm mass. However, while the biofilm score aligned with abiotic surface attachment, it did not reliably capture biotic cellular adherence to Hep-2 cells, indicating that the scoring system is primarily reflective of abiotic, PIA-dependent biofilm mass rather than host-cell adherence. All data are summarised in Supplementary Table S6.

**Fig. 4:**
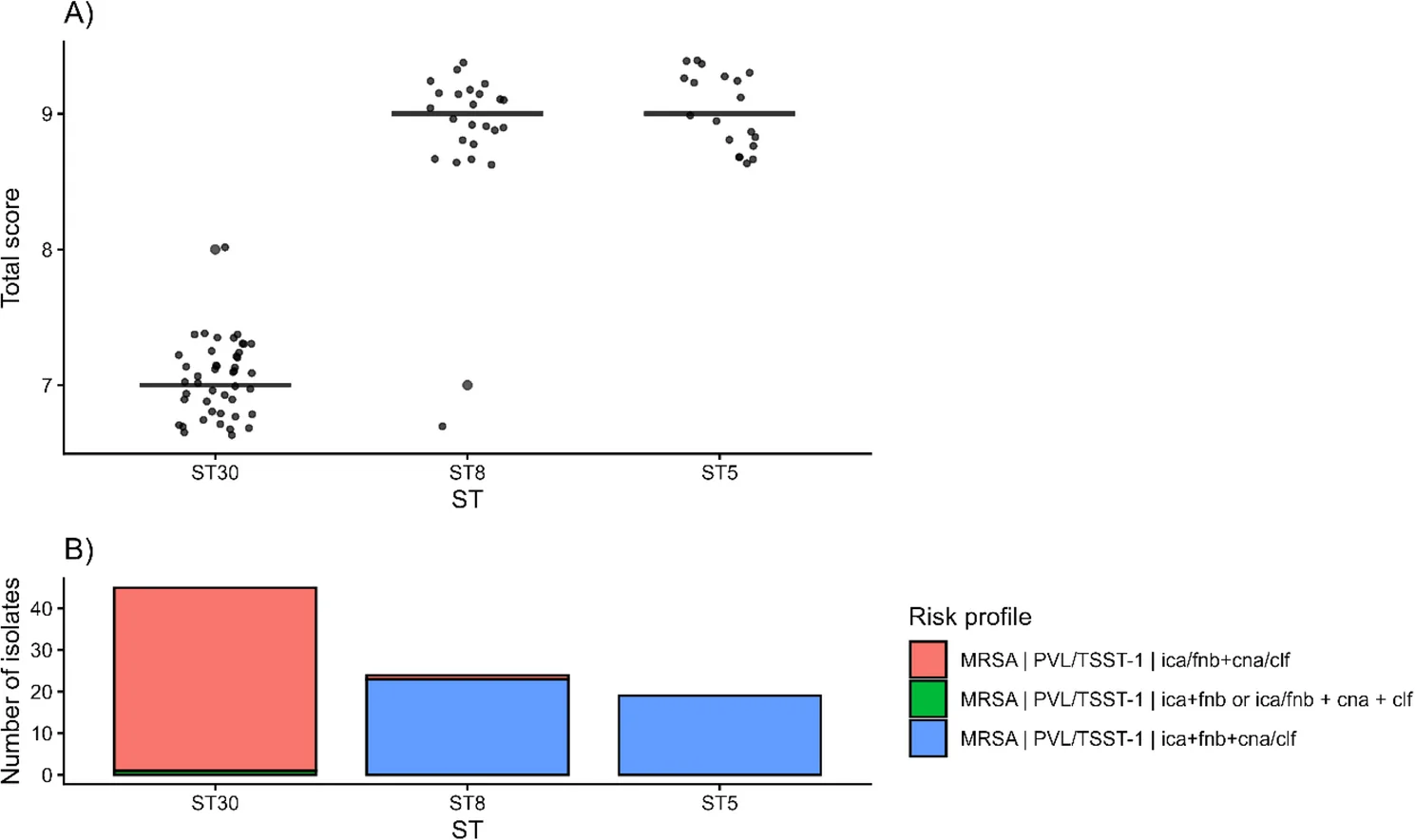
High risk isolates. Only isolates with a resistance score ≥ 2, virulence score of 3 and biofilm score ≥ 2 have been included within the top 5 STs population **A** Frequency of high-risk isolates by lineage. The total score has been calculated by summing the resistance, virulence, and biofilm scores **B** Box plot represents prevalence of high-risk clones per ST, stratified by their genotype

These scores facilitate downstream analysis, including identification of high-risk strains. We have evaluated the prevalence of high-risk strains among the top 5 STs of the dataset, defined as isolates with a resistance score ≥ 2, virulence score of 3, and a biofilm score ≥ 2. The 35.8% (88/243) of the isolates have a high-risk genomic profile, all belonging to the lineages ST30, ST5, and ST8. The ST30 was the most frequent, accounting for the 51.7% (45/88), with ST5 and ST8 having the isolates with the highest total score (total score = 9) (Fig. 4). ST8 isolates in the dataset exhibit the highest risk profile, with the 40% (24/58) of the genomes being multidrug resistant (MDR), PVL/tsst-1+, with high biofilm-producer potential.

### Comparative analysis and benchmark

A comprehensive comparison between StaphSCAN and StaphScope [9] is reported in Table 4. While both tools provide core epidemiological typing for *Staphylococcus aureus*, their underlying design differ significantly. StaphSCAN incorporates several specialized typing modules absent in StaphScope [9], including agr typing, capsular typing, and a dedicated biofilm module. Furthermore, StaphSCAN automatically excludes non-aureus genomes during the identification step and provides high-resolution subtyping for SCCmec elements. Conversely, StaphScope [9] uniquely includes a dedicated plasmid characterization module.

**Table 4:** Features’ comparison between StaphSCAN and Staphscope

Feature	StaphSCAN (v0.4.0)	StaphScope (v1.1.0)[9]
Identification	Provided (filter out not S. aureus genomes)	Not provided (no filter)
Assembly QC	Provided	Provided
MLST	Provided	Provided
SCCmec typing	Provided (including subtyping)	Provided (no subtyping)
Spa typing	Provided	Provided
Agr typing	Provided	Not provided
Capsular typing	Provided	Not provided
Biofilm-specific module	Provided	Not provided
AMR specific module	Provided	Provided
Virulence specific module	Provided	Provided
Plasmid specific module	Not Provided	Provided
Report summary	Provided	Provided

Beyond feature availability, the tools diverge in their approach to data reporting and usability. StaphScope [9] employs a “generalized” approach, reporting all identified genes (both core/intrinsic and acquired) and often logging multiple hits for the same gene when querying multiple databases. While this provides exhaustive data, it generates a highly fragmented output: when used on the 404-genome dataset, StaphScope [9] generated 18,226 total files distributed across 9 different folders, with 10 files designated as final reports.

In contrast, StaphSCAN is designed for high-throughput, standardized genomic surveillance. It employs a targeted approach, focused on clinically relevant, acquired genes. It summarizes this information using a score-based system and compiles all results into a single, unified, tab-delimited file. This simplifies data interpretation, facilitates the merging of different sequencing runs, and allows for effortless integration into broader epidemiological workflows.

Computationally, StaphSCAN demonstrated superior efficiency (Table 5). Despite both tools being accessible via Conda and requiring minimal hardware, StaphSCAN completed the analysis of all 404 genomes in 52 min utilizing only a single thread (1 CPU) and 4 GB of RAM. StaphScope [9], despite operating in a multi-threaded capacity (5 CPUs and 16 GB of RAM), required more than 3 h to process the same dataset. Moreover, running on HPC, the resource management of StaphScope [9] was always causing an out-of-memory error when using AMR module, which was skipped. Furthermore, StaphSCAN’s output disk footprint was highly optimized, requiring less than 1 MB compared to StaphScope’s ~ 300 MB. Since the OOM failure was causing to skip one of the core StaphScope module, we have compared the performance locally on a subset of 10 samples, using a laptop with 8 cores and 16 GB of RAM: StaphSCAN completed the analysis in 58 s, generating 1 output file of 12 kb, while StaphScope [9] completed the analysis in 4 min, generating 553 files and occupying 17 megabytes of disk space.

**Table 5:** Computational features of StaphSCAN vs. StaphScope

Computational features	StaphSCAN (v0.4.0)	StaphScope (v1.1.0)[9]
Installation method	Conda (Bioconda channel)	Conda (requires custom third-party channel)
Parallel processing	Single-threaded	Multi-threaded
Minimum Resource requirements	Very low (1 CPU, 4 GB of RAM)	Low to Moderate (4 + GB RAM, 2 + CPUs)^
Analysis results		
Runtime per sample	~ 5.8s*	~ 24s*
Output disk footprint	< 1 MB*	~ 17 MB
Output files count	1	553

^As reported in the relative paper

*Using the parameters reported in this paper, running on local computer with 8 cores and 16gb of RAM, on a dataset of 10 genomes

Ultimately, while StaphScope [9] offers utility for exhaustive local analysis, StaphSCAN provides a highly optimized, single-file framework essential for rapid, large-scale clinical surveillance.

StaphSCAN was also compared to Pathogenwatch (v.81) (https://pathogen.watch/en), a web-based platform run by the Centre for Genomic Pathogen Surveillance (https://www.pathogensurveillance.net/). Overall, Pathogenwatch could replicate some of the modules provided by StaphSCAN, including species confirmation, assembly quality check, mlst, and acquired resistance, including the relative antibiotic class resistance associated, but it lacks other features including spa, cap, and SCCmec typing, and the detection of virulence and biofilm genes. Given the different nature of the two, command-line vs. web-based, a computational benchmark was not possible. The main advantage of Pathogenwatch over StaphSCAN relies on being web-based, not requiring bioinformatic skills neither any infrastructure apart from an internet connection, representing a valid alternative whether not possible to run a CLI-based tool.

In this study we have presented StaphSCAN, a single framework for rapid genotyping, including species detection, sequence typing, genotyping of key antimicrobial and virulence determinants. The strength of StaphSCAN is its species-specific workflow, capable of providing only results relevant to the *Staphylococcus aureus*, reducing the noise of the most ‘general’ tools. For example, all intrinsic genes, like *tet38* or *norA*, are not analysed by StaphSCAN, since their presence is constitutive and do not provide relevant insights into acquired resistance mechanisms [43, 44]. The results are reported to make the results informative and easy to read at the same time, with the scoring system facilitating an immediate interpretation.

We successfully reproduced the results of a large, international and multi-center study involving more than 400 isolates originally requiring a combination of several tools, just using StaphSCAN. Using the score system, we could also provide novel insights on the prevalence of high-risk strains among the most prevalent lineages in the dataset.

### Limitations

StaphSCAN has several limitations to consider. It is a strictly species-specific pipeline; while this enables highly tailored and rapid analysis, it provides insights exclusively for *Staphylococcus aureus*, limiting its utility in broader, multi-species genomic surveillance. Moreover, while StaphSCAN includes a rapid quality check, it is fundamentally designed as a downstream typing tool. It therefore requires prior computational steps, specifically the generation and a rigorous quality check of assemblies, and more comprehensive upstream quality control, including read-level taxonomic profiling to rule out contamination. The underlying databases rely on manual curation, which may result in slight delays when incorporating newly discovered alleles or resistance determinants. While the scoring system (Resistance, Virulence, and Biofilm) is valuable for summarizing complex genomic data into easily interpretable profiles, these scores merely reflect the genotypic detection of specific determinants. They represent a presumptive clinical risk and must not be used as direct, definitive predictors of in vivo clinical outcomes or disease severity. Finally, the validation included in this study is mostly based on WGS-based approaches only, which may limit actual accuracy validation.

## Conclusions

In conclusion, we have developed StaphSCAN, an open-source, lightweight command-line tool tailored for the genomic surveillance of *Staphylococcus aureus*. As whole-genome sequencing becomes a routine diagnostic standard, the primary bottleneck in public health has shifted from data generation to data interpretation. StaphSCAN directly addresses this by translating complex genomic assemblies into simple, specific, and clinically actionable information, with an intuitive scoring systems to rapidly flag high-risk isolates based on their antimicrobial resistance, virulence, and biofilm potential. Looking forward, the true utility of StaphSCAN lies in its seamless integration into broader epidemiological and clinical surveillance frameworks. Because of its extremely low computational footprint and standardized, single-file tabular output, it is an ideal downstream typing module for automated, high-throughput bioinformatics ecosystems. This is already underway, as evidenced by StaphSCAN’s recent incorporation into the nf-core framework and the Nextflow-based Bactopia pipeline. At the local hospital level, StaphSCAN outputs can be easily parsed by Laboratory Information Management Systems (LIMS) to rapidly alert infection control teams to ward-level outbreaks or hypervirulent clones (e.g., PVL/TSST-1 positive MRSA). On a national or global scale, its standardized reporting format facilitates data harmonization across multi-center networks, empowering resource-limited settings to participate in real-time genomic epidemiology.

Future development directions for StaphSCAN will focus on addressing current bioinformatic limitations and expanding its predictive scope, including the support for raw read-level analysis, bypassing the assembly bottleneck entirely.

## Availability and requirements

Project name: StaphSCAN

Project home page: https://github.com/riccabolla/StaphSCAN

Operating system(s): Linux, macOS, and Windows (via Windows Subsystem for Linux). 

Programming language: Python3

Other requirements: mash (v2.0+), blast, biopython (v1.78+), pandas, rauth, and requests. 

License: MIT

Any restrictions to use by non-academics: None

## Supplementary Information

Supplementary Material 1.

Supplementary Material 2.

## Data Availability

The complete source code is available on GitHub under a MIT license: https://github.com/riccabolla/StaphSCAN. A documentation is available at: https://staphscan.readthedocs.io/en/latest/. The tool version and the databases used in this manuscript are hosted at Zenodo: https://doi.org/10.5281/zenodo.20932930. The public dataset used are available at PRJEB37318 and PRJNA890445. For dataset PRJEB37318 individual accession numbers are available in Supplementary Table S1.
