# Supplementary material for: StaphSCAN: a genomic surveillance framework for Staphylococcus aureus
Source: BMC Genomics. 2026 Jul 9;27:807. doi: 10.1186/s12864-026-13165-0 (PMC13632141; doi:10.1186/s12864-026-13165-0)
Supplement: Supplementary file 1 [file 12864_2026_13165_MOESM1_ESM.docx]

Figure S1. Distribution of pairwise Mash distances between genomes of different species and *Staphylococcus aureus* genome in the reference database. The number of genomes is shown on the y-axis and distance on the x-axis.
